# Dr. Stewardson on Remittent Fever

**Published:** 1842-04-16

**Authors:** 


*Observations on Remittent Fever, founded upon Cases observed in the Pennsylvania Hospital.* By Thomas Stewardson, M. D., one of the Physicians to the Institution. [*Am. Jour. Med. Scien.*, *April*, 1842.]

We gave some account at the time of its publication, of the first part of this memoir; it is concluded in the present memoir, which includes the history of the symptoms and treatment. We shall extract all the conclusions which the author has given us.

The *tongue* was covered with a thick yellow fur in two-thirds of the cases; with this exception, the tongue presented no important alteration, except in two or three cases—a result very different from that observed in typhus and typhoid fevers. A similar difference exists as to the time of cleaning. In eight cases of eleven the tongue began to clean on or before the eighth day; from the thirteenth to the twentieth in the remaining three. The *thirst* was generally moderate, except during the exacerbations.

"Pain or tenderness at the epigastrium or hypochondrium, or both, were present in every case but one." Local abstractions of blood were often necessary. In typhoid fever pain is felt in this situation in less than half the cases; a difference sufficiently accounted for by the different situation of the abdominal lesions.

*Vomiting* of greenish matter was very frequent, while the bowels were generally costive.

The *pulse* was not very frequent, except during the exacerbations. We have often found it in certain epidemics preternaturally slow and weak.

"*Chills and Remissions.*—In the cases where this circumstance has been noted, the attack was usually ushered in by a chill, which sometimes did not amount to more than a sensation of coldness. In two cases, however, if the patients were correct in their statements, the chill came on subsequently to the commencement of the attack; in one case, on the day following, and in the other three or four days afterwards. In one case only, is the absence of chill throughout the whole course of the disease positively noted. The recurrence of the chill was subject to great diversity; either there were none after the first, or they recurred at intervals, most commonly of twenty-four or forty-eight hours for the first few days, and then disappeared altogether, or again reappeared towards the conclusion, or during convalescence, or finally showed themselves at various intervals throughout the whole course of the disease. During the time in which the patients were the subjects of observation in the Hospital, the chill, or sensation of coldness, when it did recur, was found to do so generally after an interval of forty-eight hours, or, in other words, the type of the disease was evidently tertian. In exemplification of this and some of the other points just referred to, as well as affording examples of the character and succession of the symptoms in simple remittent, I shall give in detail the history of two out of three cases, which occurred in individuals who had arrived in the same vessel, and where the disease commenced and terminated almost simultaneously."

The temperature of the skin varied greatly with the exacerbations. No rose-coloured spots were found,--we have always looked for them in vain. Sudamina were observed in several cases. They were probably much more frequent, but they disappear so rapidly that it is often difficult to find them. The colour of the skin was not always noted. It was yellowish or sallow in most cases, bluish or white in two cases of malignant remittent. This coincides with our own observations; the natural tint of a patient affected with remittent is yellowish, unless the fever be of the malignant, or, as it is often called, the congestive variety.

The affections of the chest, it is well known are insignificant in remittent fever. Those of the cerebral organs are much more frequent; headache is almost an invariable attendant, just as it is in other diseases of febrile action. The delirium was very mild in the cases of remittent fever observed by Dr. Stewardson, even when of the congestive form. This coincides with general observation; but there is a variety of remittent attended with a jaundiced complexion, in which the delirium is severe and long continued. On the whole, however, delirium is much more manageable in remittent than in typhoid or typhus fever.

The senses were but slightly affected in the cases described by Dr. S.; subsultus, which is almost always present in typhus and very often in typhoid fever, was observed in one case only.

Dr. Stewardson found that in sixteen of the seventeen favourable cases, the average duration of the disease was seventeen and a quarter days; striking out two cases under peculiar circumstances, he found that the mean duration was reduced to fifteen days, which we believe with him to be about the ordinary duration.

The mortality was insignificant in the milder cases admitted into the hospital. Many, however, were severe, giving a mortality for the whole of one in ten and a half.

Dr. Stewardson has given some interesting remarks relative to the diagnosis of yellow and remittent fever; practically, however, the diseases can scarcely be confounded together, except in epidemics of yellow fever. Even then a little care and attention to the remittent character of the proper remittent fever, and the single paroxysm of yellow fever, will in most cases remove the difficulty. The distinction between remittent and typhoid fever is so obvious, that if attention be paid to the commencement and progress of the two diseases, they can scarcely be mistaken; if they be compared, however, in certain stages, without reference to the previous history, it is often difficult to distinguish them.

The nature of the disease is ascribed by Dr. Stewardson to the alteration of the blood, and to certain disorders of viscera combined with it; in this respect pathologists generally agree. The nervous system, especially the spinal marrow, however, plays an important part in all paroxysmal forms of fever. The triple lesion, of the liver spleen and stomach of remittent, as contrasted with the triple lesion of the small intestines, mesenteric glands and spleen of typhoid fever, and the humoral alteration of typhus, we had occasion to insist upon in a memoir published some five years since.

"*Treatment.*—General bleeding was not often resorted to. Indeed, comparatively few of the patients were admitted at that early stage of the disorder, when, according to the experience of some of the best practical writers, it is particularly useful, or in severe cases, even safe. Topical bleeding, on the contrary, by cups or leeches to the head and epigastrium, was often resorted to. To the latter region they were applied, especially in cases where there was considerable pain and tenderness, and for the most part with the most decided benefit to these, as well as the other gastric symptoms. Besides this, when the irritation of the stomach was considerable, the patient was confined to cold, acidulated drinks, as barley water with lemon juice, the effervescing draught, &c. Unless in cases, and they were not frequent, where there was already some diarrhœa, the bowels were freely evacuated by a brisk purgative, such as the sulphate of magnesia, alone or with senna. Even in typhoid fever, where the condition of the small intestine is such as has been supposed by many to contra-indicate the use of purgatives, the propriety of having recourse to them, is now advocated by some of those who formerly proscribed them; and the groundlessness of the fear entertained in reference to their deleterious effects admitted perhaps by almost all. But however plausible the objection to their use in typhoid fever, the same cannot be urged as regards remitting fever; for here, unless where there is some accidental complication, the small and large intestines present no evidences of inflammation, and on the contrary, are quite healthy. Reasoning *à priori*, indeed, from the condition of the organs, we should infer that a revulsive and depletory action, exercised upon the mucous membrane of the alimentary canal, must be highly beneficial in relieving the inflammatory or congested condition of the stomach, spleen and liver, and the probable overloaded condition of the portal circulation; and experience, I think, is in accordance with this view. Of the peculiar advantages of calomel as a purgative, I cannot speak, having rarely employed it, being perfectly satisfied that it is liable to great abuse, and that in the ordinary forms of the disease in this climate, its use is, at any rate, not especially demanded. Small doses of blue pill, however, combined with a few grains of rhubarb, and repeated every three or four hours, I have frequently used with advantage in cases of some severity, where it appeared desirable to keep up a moderate and uniform action upon the bowels. Generally, however, after free discharges had been once procured, the employment of neutral mixture, or some similar remedy, was all sufficient, keeping up a slight action upon the bowels, at the same time that it operated favourably as a diaphoretic. But while moderate purging is eminently serviceable, we should carefully avoid bringing on an irritated condition of the alimentary canal by the frequent repetition of active cathartics. In a few instances, there is a strong tendency to this condition, so that even the mildest articles cannot be borne; but as a general rule, hypercatharsis has not resulted from the moderate use of purgatives.

The topical depletion to the epigastrium, already alluded to, (the blood was generally drawn by means of cups,) was repeated several times at various intervals, according to circumstances, and as before remarked, was most efficacious in relieveing the pain and distress, as well as the irritability of stomach in cases where this was present. The cups also were frequently extended across the hypochondria, especially the left. The amount of blood drawn was commonly not large, from four to eight ounces, and was proportioned of course to the degree of febrile reaction and the strength of the patient. It was during the period of exacerbation, or when the pulse was accelerated, and the skin warm and dry, that this measure was chiefly demanded, as the local symptoms were then most marked. It is also more than probable that, employed at this period, its permanently salutary influence upon the viscera was best attained. In the violent remittents of the East Indies, it is said by Dr. Twining to be a dangerous practice to abstract blood as the febrile paroxysm is passing off, and after perspiration has broken out, as the most alarming and even fatal prostration sometimes results; and although this effect be much less likely to occur in the more ordinary remittents of temperate climates, it should always be borne in mind that the most proper period for depletion is during the exacerbation and before perspiration has come on. The rapid changes which take place in this disease, render it peculiarly important for the practitioner to be upon his guard and direct his remedies accordingly. As regards the stage of the disorder, I should say that it was not worthy of much consideration in determining upon the propriety of local depletion in cases of our ordinary remittent, where considerable epigastric or hypochondriac tenderness coincided with more or less acceleration of pulse and heat of skin. For although here, as in the more severe disease of hot climates, early depletion, *i. e.* from the first to the fourth day, is highly desirable, in order to shorten its course and diminish the force of local determinations, yet the same danger does not exist as in the latter, in reference to depletion at a much later period, unless of course where the symptoms of prostration clearly forbid it. I would not hesitate then to abstract a few ounces of blood under the circumstances mentioned, even at a late period of the disorder, since it is certainly a point of paramount importance in the treatment of remittent, to prevent, as far as practicable, the production of those chronic alterations of the spleen and liver, which, when once firmly rooted, so generally prove fatal after lengthened suffering. To accomplish this end, as well as to relieve the febrile and gastric symptoms for the time, in cases where early and vigorous depletion has not been resorted to, or where these have not been sufficient, the local abstraction of blood, as above mentioned, even in small quantity, and repeated, if necessary, during several successive exacerbations, is one of the most effectual means. Still, however, it should be recollected, that even with us, cases of a less inflammatory character are met with where the pulse is soft or compressible, and where the loss of even a small quantity of blood may produce the most alarming symptoms of prostration; and where, of course, if resorted to at all, it should be done with great caution, and its effects carefully watched. The local abstraction of blood from the temples and back of the neck was often resorted to with the most decided benefit, especially during the violence of the exacerbation, to relieve the determination to the brain, as indicated by heat of head, cephalalgia, accompanied, perhaps, by wandering and delirium: cooling lotions, &c. to the head of course concur to the same end. These remedies, together with some mild diaphoretic, as the neutral mixture already mentioned, the spiritus mendereri, &c. were, in the majority of cases, sufficient, until that period when a complete and full remission of the symptoms took place. Sometimes, however, considerable stupor, or even profound coma comes on, and here, if the condition of the patient will admit of it, blood should be drawn from the temples, or back of the neck, and a brisk cathartic administered. It is here that I have used with advantage a large dose of calomel, followed, however, by some other cathartic, as senna and salts. A blister to the back of the neck is also highly important. Sometimes also, in the latter stage of the disease, the tongue becomes dry or even brown, the remissions very imperfect, and the aspect of the patient assumes more or less of a typhoid character; and here, as in typhoid fever, properly so called, small doses of blue pill alone, or combined with opium or rhubarb, according to circumstances, are especially useful.

Having, by the measures already indicated, reduced the inflammatory condition of the system, and a free remission of the symptoms having taken place, the pulse becomes soft, and the skin perspirable, the sulphate of quinine, to the amount of from ten to fifteen grains, was usually administered with the most decided advantage. That, given in this way, quinine exercises a control over remittent very similar to that which it does over intermittent, either entirely preventing, or very much modifying the subsequent exacerbation, and by its further repetition entirely arresting the disease, I am firmly convinced. Much difference of opinion has existed among medical men in reference to the propriety of employing the various preparations of bark, some extolling them highly, whilst others proscribe them altogether. Such discrepancy among men of acknowledged merit, can hardly be accounted for, without supposing that the effects of the remedy are very different in different forms of the disease, at different seasons, &c. Thus, in the season of 1839, in Philadelphia, according to the experience of more than one practitioner, the disease, after a few days of preliminary treatment, the remissions generally being very perfect, was promptly controlled by quinine, more so than in ordinary years; whilst on the other hand, during the past season, it seems to have been influenced much less favourably by the remedy, which in some cases could not be borne. Undoubtedly also, in comparing the experience of the last few years with that of a more remote period, we should recollect, that the preparations generally made use of are very different, and that the sulphate of quinine might be given safely and in sufficient quantity during the remission to produce the most decided beneficial effects in cases where the crude bark formerly used, might be useless or absolutely hurtful. Be this, however, as it may, the value of quinine in the treatment of remittents, appears to be every day advancing in the estimation of practitioners in different parts of the United States. Whether it will completely arrest the disease at a very early stage, at least as a general rule, is very doubtful. It is especially at a later period, after the use of depletory and other measures, and when a complete remission has taken place, that its effects are most decided. Still, there is reason to believe, that at an early period, if cautiously administered during the remission, its influence upon the future course of the disease is highly favourable. Undoubtedly a large proportion of cases may be safely treated without this remedy, but there are others again in which I think that it is imperatively demanded. These cases, I should say, were particularly characterized by softness of the pulse and a tendency to prostration; and here I have little question that by a neglect of the remedy, disastrous results would sometimes take place which might otherwise have been prevented. Besides the conditions already mentioned, the administration of quinine is important, as convalescence approaches in all forms of the affection, with a view to prevent its degeneration into irregular intermittent. Of course, in the remarks which I have hazarded upon this point, as well as in the description of the general treatment, I have reference mainly to the ordinary form of the disease as it has presented itself to my observation. In the high grades of bilious remittent, I have had but little opportunity of forming an opinion as regards the effect of remedies. Of the efficacy of quinine in pernicious remittent, as well as of the importance of administering it promptly and in large doses, there can scarcely be a question; and where the danger was imminent, I have not hesitated to continue its use, even through the exacerbations, as in a case mentioned, where the pulse was soft and the reaction imperfect.

In the administration of quinine, I have usually employed the solution, with a few drops of elixir of vitriol, in which form it is, perhaps, most prompt in its operation. The dose was commonly from four to eight grains, repeated according to circumstances. Sometimes given alone, it was at others combined with sweet spirits of nitre and laudanum.

I have already spoken of the efficacy of blisters to the back of the neck in cases where there was great stupor. Their application to the epigastrium also in cases where there is much præcordial pain, uneasiness or oppression, or irritability of stomach, accompanied with more or less prostration or coolness of the surface, is a point of practice never to be lost sight of. In the more inflammatory cases, however, they may prove more annoying than useful, and besides, the local abstraction of blood from the epigastrium is here preferable, and generally sufficient."

Instead of confining ourselves to mere extracts from this paper, we have placed our readers in possession of all the important matter contained in it, not only on account of its intrinsic value, but from our knowledge of the care and ability with which the author conducted his inquiries.

